# Prevalence, Causes, and Risk Factors of Visual Impairment in Emiratis and Non-Emiratis of Dubai: A Subnational Population-Based Cross-Sectional Survey

**DOI:** 10.1155/2022/9726230

**Published:** 2022-04-30

**Authors:** Manal O. Taryam, M. Mansur Rabiu, Shurooq AlBanna, Noora Al Shamsi, Bushra Albastaki, Hayat Khan, Salam Chettiankandi, Wafa Khamis Alnakhi, Hamid Y. Hussain, Prasan Rao, Gurdeep Singh, Sivakami Pai, Mazen M. Sinjab, Lama Toufik Sharbek, Xianwen Shang, Mingguang He

**Affiliations:** ^1^Noor Dubai Foundation, Dubai Health Authority, Dubai, UAE; ^2^Dubai Health Authority, Dubai, UAE; ^3^Medcare Hospital and Clinics, Dubai, UAE; ^4^Al Zahra Hospitals, Dubai, UAE; ^5^Medcare Eye Centre, Dubai, UAE; ^6^Centre for Eye Research Australia Ltd, University of Melbourne, Australia

## Abstract

**Purpose:**

To study the prevalence, causes, and risk factors of visual impairment (VI) among the Dubai Emiratis and non-Emiratis.

**Methods:**

The survey was a population-based cross-sectional eye health study conducted 2019-2020. Cluster sampling was used to randomly select local (Emirati) and expatriate (non-Emirati) Dubai residents aged 40 years and older. Ocular examinations were conducted in selected eye clinics to determine the visual acuity (VA) and cause(s) of VI if any. Trained nurses, optometrists, and ophthalmologists did the examinations. VA was measured using ETDRS visual chart. The World Health Organization VI and blindness definitions and classifications for the cause(s) of VI were used.

**Results:**

A total of 892 participants were included in the final analysis. The mean age [SD] was 52.09 [9.48] years, with 55.8% as males. Prevalence of presenting mild, moderate, and severe VI was 4.7% (2.94–7.11%), 1.8% (0.78–3.5%), and 0% for Emiratis, and 3.6% (2.06–5.76), 1.6% (0.63–3.21), and 0% for non-Emiratis, respectively. Four Emirati participants were blind, with a prevalence of 0.9% (0.25%–2.28%). Men had lower likelihood of VI than women (odds ratio [OR] (95% CI): 0.42 (0.24–0.77)) after adjustment for covariates. Diabetes (OR (95% CI): 1.91 (1.04–3.52)) was an independent risk factor for VI. Higher education level was associated with a lower likelihood of VI (OR (95% CI): 0.34 (0.13–0.89). Leading causes of VI among Emiratis were uncorrected refractive error (52%) and cataract (17.2%). Glaucoma, optic atrophy, and absent globe were the causes of blindness.

**Conclusions:**

Prevalence of VI is comparably low with leading causes readily treatable. An effective strategy to improve spectacle correction and cataract services would reduce the VI burden.

## 1. Introduction

Visual impairment (VI) and blindness are important global health problems, leading to increased mortality, reduced quality of life, and significant economic loss [1,2]. The latest Global Burden of Disease Study (GBD) estimates 43.3 million people who were blind, 295 million had moderate and severe VI, and 258 million had mild VI worldwide in 2020 [3]. The number of people with VI and blindness from 2020 to 2050 is estimated to increase by approximately 90% and 50%, respectively [3]. Although the age-adjusted prevalence of VI and blindness had decreased over the past three decades, the absolute number of people with VI and blindness continues to increase. This suggests that progress in disease control is not keeping pace with demand, and more challenges are expected with the continued rapid global population growth and aging [4]. In 2019, the World Health Organization (WHO) launched the first “world report on vision” to draw attention to the increasing need for eye care, and called for coordinated, concerted global action toward strengthening eye care in health systems and collecting key indicators for eye health in member states [5].

Reduction in the prevalence of VI and blindness over past decades can be attributed to the VISION 2020—the Right to Sight initiative [6]. Most countries worldwide including the United Arab Emirates (UAE) have signed formal declarations of support for this global initiative, which requests countries and stakeholders to establish national eye care programs and monitor the burden of VI and blindness. The WHO has consistently emphasized the need for population-based data from all countries, to better estimate VI-related burden, and inform resource allocation and best practice patterns [5–8]. However, such studies have been conducted in less than 20 percent of countries globally [9].

There is a dearth of data on the prevalence and causes of VI in the Dubai Emirates as well as the whole country of UAE. The only existing survey regarding VI prevalence from the UAE is a hospital-based study from the city of Al-Ain, in the Emirates of Abu-Dhabi [10]. Lack of population-based data on VI and eye diseases is a serious impediment to effective national planning of eye care programs in Dubai Emirates and non-Emirates. We, therefore, conducted a population-based survey to gather population-level data on prevalence, causes, and risk factors of VI in the Dubai Emirates and non-Emirates.

## 2. Materials and Methods

Dubai is the second largest of the seven Emirates that make up the UAE. It occupies 4,114 square kilometers [11]. In 2016, it had an all-age estimated population of 2,504,000. Dubai's population age and gender ratios are skewed, with approximately 75% of the population being male and 58% of the population in the 25–44 age group [12]. Dubai has a unique population structure where only 15–20% of the population are natives, with the remaining 80% composed of expatriates, who are mostly single young adult males [13].

### 2.1. Sample Size

A sample size of 2190 was calculated using the following formula [13]:

(1) $$\begin{array}{c} N=\frac{Z2^{*}\left(p^{*}q^{*}\text{deff}\right)}{\left(e^{*}1/RR\right)}=2190. \end{array}$$

where

- *z* = 1.96, statistics from standard normal distribution for 95% confidence level.
- *p*=0.05, proportion of VI in Dubai population 40+ years of old, according to Disability Survey in Dubai 2018, and q = 1-p = 0.95.
- deff = 1.5, design effect for cluster survey.
- RR = 0.80, response rate.
- *e* = 0.0125, margin of error.

### 2.2. Study Design

The study is a population-based cross-sectional survey of residents aged 40 years and older residing in Dubai conducted between December 2019 and March 2020. Residents were defined as persons that had lived in that household in the last six months.

The study population was divided into two main categories. The Emiratis are the nationals of UAE and non-Emiratis are expatriates. The expatriates were further stratified into three groups, namely, professionals (professional expatriates), collective households (the blue-collar expatriates), and labor camps (laborers). These strata have different demographics and accessibility to eye care services. Based on the proportion of 40 years and older and the assumed proportion of people with VI in each stratum, the sample size was shared into the 4 population strata. For each stratum, a specified number of clusters were allocated and randomly selected by probability proportional to size. All 165 clusters were selected across the four population strata. In each selected cluster, households were randomly chosen from the database of residents with the Dubai Statistics Center, to yield the needed sample size for the stratum. In each selected household, one eligible person was randomly selected. Selected individuals were contacted via phone and invited to participate in the survey by visiting the nearest of the four eye clinics selected for the survey. Civil and religious societies were involved in encouraging the selected individuals to participate in the survey.

The survey was conducted by six teams and planned to run for six months beginning from December 2019. Each survey team consisted of an ophthalmologist, an optometrist, and a nurse. Survey teams were trained for 4 days on all aspects of survey examination procedures, use of the equipment, and operational definitions. An interobserver agreement of at least 80% was achieved among teams for the measurements of visual acuity (VA), and principal cause(s) of VI.

Ethical approval for the survey was obtained from the Dubai Scientific Research Ethics Committee of the Dubai Health Authority (DSREC-05/2019_03) and conducted following the Helsinki Declaration. Written informed consent was received from each participant.

### 2.3. Study Examinations

Participants' personal, demographic, and health data, including age, sex, nationality, literacy level, occupation, working status, and history of diabetes mellitus, were obtained. Random blood sugars were tested via a point-of-care BioHermes HBA1c device (BioHermes; Jiangsu, China). The nurses obtained and recorded this information. The optometrists measured the VA using the EDTRS LogMar chart at 3 meters (Tumbling E-Series ETDRS-Chart 1-3cht1-3 Meter by Precision vision; La Salle, USA) in well-lit rooms. The chart was placed one meter above the ground. The participant scores the smallest visual acuity level when he/she indicates correctly the orientation of at least three of the four characters in the level. Each eye was first tested individually without aid and recorded as “uncorrected visual acuity.” The VA was then tested with glasses/contact lenses (distant) if normally worn, for each eye, and recorded as “presenting visual acuity” (PVA). After refraction, the best-corrected VA was also tested in each eye and recorded as “best-corrected VA.” If any of the eyes did not see any letter from the chart, then VA was assessed through counting finger down at one meter to light perception.

All participants had automated refraction performed by the optometrists using the Topcon KR1 auto-refractometer (Topcon, Tokyo, Japan). Following refraction, participants with PVA less than 6/12 in any eye received subjective refraction. Participants for whom automated refraction could not be obtained due to media opacities received subjective refraction.

Detailed anterior and posterior segment assessments of both eyes were performed for all study participants by the ophthalmologists, with slit-lamp biomicroscopy, direct and indirect ophthalmoscopy (Keeler-USA), and intraocular pressure check (Goldman applanation tonometer) to determine the cause(s) of VI in any eyes, if present.

### 2.4. Study Definitions

#### 2.4.1. VI and Blindness Definition for the Eye or Person

Definitions of bilateral VI and blindness followed the WHO classification [14]: (1) normal = PVA ≥6/12 in that eye or the better eye; (2) mild VI = PVA <6/12 but ≥6/18 in that eye or the better eye; (3) moderate VI = PVA <6/18 but ≥6/60 in that eye or the better eye; (4) severe VI = PVA <6/60 but ≥3/60 in that eye or the better eye; (5) blindness = PVA <3/60 in that eye or the better eye. Unilateral VI = PVA ≥6/12 in one eye but <6/12 to ≥3/60 in the other eye. Unilateral blindness = PVA ≥6/12 in one eye but <3/60 in the other eye.

### 2.5. Diabetes Mellitus

Participants with diabetes mellitus history or HbA1c of 6.5% or greater were considered diabetic.

### 2.6. Principal Cause(s) of VI and Blindness

For all participants with VA <6/12 in either eye, the principal cause of the VI was assigned by the ophthalmologist based on all diagnostic findings. A systematic approach for determining the principal cause(s) of VI and/or blindness for each eye was established based on the WHO standardized protocol [15]. According to the protocol, the principal cause should be the pathology considered to be the most likely cause of the vision <6/12. If two different conditions in the same eye were likely to cause a degree of VI, then the condition most amenable to treatment or prevention was chosen. When more than one singular ocular disorder was present, one of which was secondary to the other, the “primary” cause was selected. For each participant with PVA less than 6/12 in one or both eyes, one single principal cause of VI or blindness was assigned for the person. If there are two different causes of VI or blindness in a person, the principal cause for the person should be the pathology most amenable to treatment or prevention.

### 2.7. Data Management and Analyses

Data were entered into a digital record form with check systems designed for the survey. Daily fieldwork data were reviewed by the survey supervisors. Statistical analyses were performed using SAS 9.4 for Windows (SAS Institute Inc.). Study participants were assigned to two groups (Emiratis and non-Emiratis) for data analyses. And three educational groups were as follows: low (illiterate), moderate (lower secondary/postsecondary education), and high levels (bachelor/masters/doctoral). Prevalence estimates were calculated and presented with 95% confidence intervals. Stepwise (forward selection) regression model was used to select covariates in the multivariable analysis. Statistical significance was assessed at *p* < 0.05 (two-tailed).

## 3. Results

**Figure 1:**
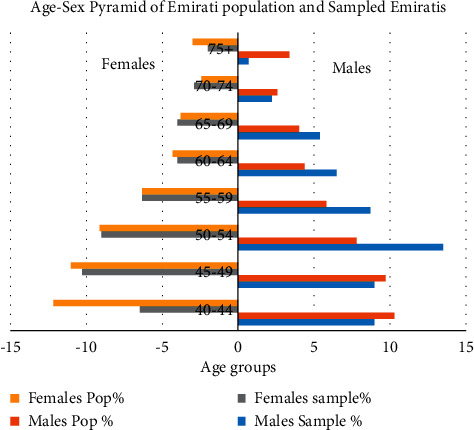
Comparison of age-sex structure of Emirati sampled participants and the general population of Emiratis in Dubai Emirate.

Of 895 participants who were examined, after excluding participants with incomplete data for vision, 892 participants (mean [SD] age, 52.09 (9.48)] years; 55.8% male, 50% Emiratis) were included in the final analysis. The age-sex structure of the Emirati sampled population depicts a more representative sample compared with the Emirati general population (Figure 1).

Demographic characteristics for Emirati and non-Emirati participants are shown in Table 1. In comparison with Emiratis, non-Emiratis were significantly younger (49.73 vs 54.42 years, *P* < 0.001), had a higher ratio of males (66.6% vs 45.1%, *P* < 0.001), and were more likely to be currently working (64.8% vs 41.5%, *P* < 0.001). Emiratis were more likely to be older than non-Emiratis (six age groups: 40–44, 45–49, 50–54, 55–59, 60–64, and ≥65 years). No significant difference between Emiratis and non-Emiratis (low (illiterate), moderate (lower secondary/postsecondary education), and high levels (bachelor/masters/doctoral), *P*=0.39).

Based on uncorrected VA, the prevalence of mild, moderate, severe VI, and blindness was 9.2%, 9.4%, 1.1%, and 1.8%, respectively, for Emiratis, and 9.0%, 7.0%, 0.9%, and 1.6%, respectively, for non-Emiratis (Table 2). While for PVA, the prevalence of mild, moderate, and severe VI was 4.7%, 1.8%, and 0%, respectively, for Emiratis, and 3.6%, 1.6%, and 0%, respectively, for non-Emiratis. With PVA, there were four Emirati participants blind with a prevalence of 0.9% (0.25% to 2.32%), while none among the non-Emiratis (Table 2). The prevalence of all categories of presenting VI (PVI) is higher in women for Emiratis (mild VI 6.1% vs 3.0%; moderate VI 2.0% vs 1.5%), and non-Emiratis (mild VI 7.4% vs 1.7%; moderate VI 2.7% vs 1.0%) (Table 2).

Projecting the prevalence values to the population of Emiratis' in Dubai, it is estimated that there could be 4174 Emiratis with all categories of VI and another 578 people blind. However, without existing/available optical corrections the number of Emiratis with VI would be almost tripled (prevalence 21.5% vs 7.4%) (Table 3).

The most common principal cause of VI among Emiratis and non-Emiratis was uncorrected refractive error (URE), followed by cataract for bilateral VI (Tables 3). URE is responsible for over 52% of the causes of bilateral VI among Emiratis. All of the VI is in the mild and moderate VI categories. Other less common causes included diabetic retinopathy, optic atrophy, and corneal opacity. For the four blind persons, the causes were glaucoma, optic atrophy, and absent globes (2 persons), all among the Emiratis (Table 3). Thirty-six Emiratis had unilateral VI, with a prevalence of 8.1% (CI: 5.6–10.7). For the non-Emiratis, it was 27 people, with a prevalence of 6.0% (CI 3.5–8.5), with the major causes being URE and cataract; others were amblyopia, glaucoma, diabetic retinopathy, and keratoconus (Table 3).

Univariate logistic regression analysis showed that participants aged 60+ years had a significantly higher risk of VI than those aged 40–49 years (odds ratio [OR] 4.37 (2.35–8.15)), and participants with higher education level had a lower likelihood of VI (moderate vs low education: OR 0.39 (0.22–0.69); high vs low education: OR 0.23 (0.09–0.57)). Participants with diabetes have a higher risk of PVI than those without OR 2.81 (1.63–4.85). In multivariable analysis, age, gender, educational levels, or diabetes status were all significantly associated with PVI (Table 4).

## 4. Discussion

This study has presented the first population-based data on the prevalence and causes of VI amongst Emiratis and non-Emiratis in Dubai, a region with a peculiar demographic structure characterized by a high proportion of the young male expatriate population. Generally, the prevalence in all categories of VI was lower in the study compared to other similar populations, even when comparing the Emirati population only [16–22]. Also, the VI was in the mild and moderate categories with the major causes being URE and cataract. It is noteworthy that although about 21% of the 40 years and older Emirati population of Dubai are projected to have some VI/blindness, after optical correction less than 1/3 of these participants remained with the VI/blindness signifying the wide coverage of the optical needs in the population. However, there is a good proportion of the population still in need of optical correction as URE still constituted over 50% of the causes of VI. Older age and lower education levels were found to be related to higher risks of VI among both Emiratis and non-Emiratis.

Previous estimates of the prevalence of VI (4.0%) and blindness (0.8%) in UAE were from a hospital-based study from the city of Al-Ain, in the emirate of Abu-Dhabi [10]. Our findings are similar to that reported from this hospital-based study but lower than the general crude global prevalence reported in the GBD study [8]. The prevalence of PVI and blindness in this study (mild VI: 4.1%, moderate VI: 1.7%, and blindness: 0.4%) was lower than that reported in several other population-based studies of similar age ranges. These include the China Nine Province Survey (mild VI: 20.4%, moderate to severe VI: 10.3%, and blindness: 1.66%), [16] the National Eye Health Survey in Australia (VI: 6.6%), [17] and the Tripura Eye Survey (VI: 8.7% and blindness: 1.5%) [18]. This could be due to the demography of our study population, with over 70% of the population being younger than 60 years due to a large number of young expatriates. This study has shown that the prevalence of VI and blindness was lower in non-Emiratis than Emiratis, possibly due to the younger demographic characteristics of the non-Emiratis.

Even when comparing our Emirati strata only (mild VI: 4.7%, moderate VI: 1.8%, and blindness: 0.9%) with other studies of similar indigenous urban populations, it showed lower VI prevalence in Emiratis. A study of adults 40 years and older in the neighboring state of Oman reported a blindness prevalence of 8.5% [19]. The prevalence of PVI in Hong Kong was 5.1%, [20] while the prevalence of VI and blindness was 11.88% and 0.73% in the National Eye Survey of Trinidad and Tobago, [21] and 7.8% and 0.2% in Singapore [22].

Consistent with previous studies, older age was a significant risk factor for VI and blindness [22–24]. Higher educational level was also found to be protective of VI and could be explained by better awareness of health status and improved financial accessibility to eye care [25,26]. Prevalence for all severities of VI has been reported to be higher in females than in males of the same age group, mainly in low-income and middle-income countries [27,28]. Consistent findings were observed in the current study although logistic regression could not confirm gender as a risk factor among the Emiratis. The factors contributing to this disparity are complex and can be attributed to both biological and social factors [27]. Major eye problems are associated with aging, with women generally having longer life expectancies, and more likely to survive to develop vision problems [28]. Life expectancy of UAE women (79.2) is higher than that of men (77.1) [29]. Gender inequalities in access to health care may also contribute to a higher prevalence of VI in females [30–32].

In line with global and regional studies, [7,8,17–22,33] URE and cataracts were the main causes of vision loss in both Emiratis and non-Emiratis in Dubai. Priority should be given to these two conditions given the reversibility and ease in treatment especially for URE, which constitutes about 50% of the causes of VI in this study. With recent increasing myopia prevalence globally, especially among younger individuals, [34] the risk of VI caused by URE may increase correspondingly in this young demographic population. In addition, the burden of cataracts and the need for effective cataract surgery are projected to increase due to increasing aging populations [35]. Government and public health agencies should therefore plan and implement strategies to address these likely future health challenges, especially the increasing health access disparity for some categories of expatriates like the laborers, a common feature for affluent communities like Dubai. However, this study could not adequately demonstrate this inequity because the study stopped before the inclusion of the large number of laborers planned to be studied.

This survey is the first population-based cross-sectional study of VI in the UAE. The cluster sampling design used ensured appropriate sampling of Dubai residents from different population groups. In addition, trained ophthalmologists, optometrists, and nurses carried out standardized examinations. The prevalence, principal cause(s), and risk factors of VI were reported for both Emiratis and non-Emiratis though the Emirati data are more reliable.

Several limitations of this current study should be noted. Firstly, due to the COVID-19 pandemic, the study had to be terminated earlier; therefore, only 895 participants (41% of the originally planned sample size) were examined. This could lead to bias in our estimates of VI prevalence. However, a comparison of the demographics of the sampled Emirati population and the general Emiratis population showed a similar pattern, which suggests estimates for the Emiratis may not be biased despite the relatively low response rate. Secondly, the sample size was calculated to detect the prevalence of VI in general, so the study may not be accurate in the estimates of the principal causes of VI. The absolute number of causes of VI was small.

In conclusion, our study is the first to provide the prevalence of VI in Dubai, a highly urbanized population with skewed demographic structure and unique challenges for conducting a population-based clinical survey. Our estimates are more reliably applied to the Emirati population, which shows a lower burden of VI, with the causes of VI being mainly avoidable. Further efforts are required to improve eye care within the community especially focused on improving refractive error services and targeted at those disproportionally affected people such as women, the elderly, and lowly educated individuals.

## Figures and Tables

**Table 1:** Characteristics of examined study participants.

Study variables	Total	Emiratis	Non-Emiratis	*P* value^*∗*^
	(*N* = 892)	(*N* = 446, 50.0%)	(*N* = 446, 50.0%)	
Age, years (mean ± SD)	52.09 (9.48)	54.45 (9.56)	49.73 (8.80)	<0.001
Age group, years^†^				<0.001
40–44	226 (25.3)	69 (15.5)	157 (35.2)	
45–49	195 (21.9)	86 (19.3)	109 (24.4)	
50–54	167 (18.7)	100 (22.4)	67 (15.0)	
55–59	111 (12.4)	67 (15.0)	44 (9.9)	
60–64	76 (8.5)	47 (10.5)	29 (6.5)	
≥65	117 (13.1)	77 (17.3)	40 (9.0)	
Gender				<0.001
Female	394 (44.2)	245 (54.9)	149 (33.4)	
Male	498 (55.8)	201 (45.1)	297 (66.6)	
Education level				0.39
Illiterate	58 (6.5)	39 (8.7)	19 (4.3)	
Lower secondary/postsecondary	639 (71.6)	315 (70.6)	324 (72.6)	
Bachelor/masters/doctoral	195 (21.9)	92 (20.6)	103 (23.1)	
Working status				<0.001
Currently working	474 (53.1)	185 (41.5)	289 (64.8)	
Not working	418 (46.9)	261 (58.5)	157 (35.2)	
Diabetes				<0.001
No	632 (70.9)	285 (63.9)	347 (77.8)	
Yes	260 (29.1)	161 (36.1)	99 (22.2)	

^*∗*^ *T*-test was used to test the difference in continuous variable between Emiratis and non-Emiratis and the chi-squared test for categorical variables. ^†^All these data are frequency (percentage).

**Table 2:** Prevalence of uncorrected and presenting visual impairment/blindness among Emiratis and non-Emiratis by sex.

Category	Emiratis (*n* = 446)							Non-Emiratis (*n* = 446)						Total
	Uncorrected VI, no. (%)			Presenting VI, no. (%)				Uncorrected VI, no. (%)			Presenting VI, no. (%)			Presenting VI
	Males	Females	Total	Males	Females	Total	Magnitude among Emirati population	Males	Females	Total	Males	Females	Total	All sexes, percentage (95% CI)
Mild VI (<6/12,6/18)	14 (7.0)	27 (11.0)	41 (9.2)	6 (3.0)	15 (6.1)	21 (4.7)	3018	25 (8.4)	15 (10.1)	40 (9.0)	5 (1.7)	11 (7.4)	16 (3.6)	4.1 (2.9–5.7)
Moderate VI (<6/18,6/60)	22 (10.9)	20 (8.2)	42 (9.4)	3 (1.5)	5 (2.0)	8 (1.8)	1156	16 (5.4)	15 (10.1)	31 (7.0)	3 (1.0)	4 (2.7)	7 (1.6)	1.7 (0.9–2.8)
Severe VI (<6/60,3/60)	0	5 (2.0)	5 (1.1)	0	0	0	0	3 (1.0)	1 (0.7)	4 (0.9)	0	0	0	0
All VI (<6/12,3-60)	36 (17.9)	52 (21.2)	88 (19.7)	9 (4.5)	20 (8.2)	29 (6.5)	4174	44 (14.8)	31 (20.8)	75 (16.8)	8 (2.7)	15 (10.1)	23 (5.1)	5.8 (4.3–7.4)
Blindness (<3/60)	5 (2.5)	3 (1.2)	8 (1.8)	3 (1.5)	1 (0.4)	4 (0.9)	578	7 (2.4)	0	7 (1.6)	0	0	0	0.4 (0.1–1.1)
Total (VI and blindness)	41 (20.4)	55 (22.4)	96 (21.5)	12 (6.0)	21 (8.6)	33 (7.4)	4752	51 (17.2)	31 (20.8)	82 (18.4)	8 (2.7)	15 (10.1)	23 (5.2)	6.3 (4.7–7.9)

VI = visual impairment; CI = confidence interval.

**Table 3:** Principal causes of bilateral and unilateral visual impairment/blindness among Emiratis and non-Emiratis

Principal cause	Emiratis (*n* = 446)							Non-Emiratis (*n* = 446)						
	No. (%) of persons by level of VI/blindness					Unilateral VI/blindness		Bilateral VI/blindness					No. (%) of persons by level of VI/blindness	
	Mild VI	Moderate VI	Severe VI	Total VI <6/12-3/60	Blindness	Total VI <6/12-3/60	Blindness	Mild VI	Moderate VI	Severe VI	Total VI <6/12-3/60	Blindness	Total VI <6/12-3/60	Blindness
Cataract	1	4	0	5 (17.2)	0	10 (27.8)	0	5	1	0	6 (26)	0	7 (25.9)	0
Refractive error	12	3	0	15 (52)	0	14 (46.7)	0	10	5	0	15 (65)	0	7 (25.9)	0
Age-related macular degeneration	0	0	0	0	0	1 (4.8)	0	1	1	0	2 (8.7)	0	0	0
Post capsule opacification	0	1	0	1 (3.4)	0	1 (2.8)	0	0	0	0	0	0	0	0
Corneal opacity	2	0	0	2(6.9)	0	1 (2.8)	0	0	0	0	0	0	1 (3.7)	1 (33.3)
Keratoconus	1	0	0	1 (3.4)	0	0	0	0	0	0	0	0	2 (7.4))	0
Glaucoma	0	0	0	0	1 (25)	2 (5.5)	0	0	0	0	0	0	0	0
Optic atrophy	2	0	0	2 (6.9)	1(25)	1 (2.8)	0	0	0	0	0	0	0	0
Diabetic retinopathy	2	0	0	2 (6.9)	0	2 (5.5)	0	0	0	0	0	0	1 (3.7)	0
Macular degeneration	0	0	0	0	0	1 (2.8)	1 (100)	0	0	0	0	0	0	1 (33.3)
Amblyopia	0	0	0	0	0	3 (8.3)	0	0	0	0	0	0	5 (18.5)	0
Absent globe	0	0	0	0	2 (50)	0	0	0	0	0	0	0	0	0
Anterior uveitis	0	0	0	0	0	0	0	0	0	0	0	0	0	1
Others	1	0	0	1 (3.4)	0	0	0	0	0	0	0	0	4 (14.8)	1 (33.3)
All	21	8	0	29 (100)	4 (100)	36 (100)	1 (100)	16	7	0	23 (100)	0	27 (100)	3 (100)

VI = visual impairment.

**Table 4:** Multivariable logistic regression analysis for risk factors associated with presenting vision impairment (visual acuity <6/12) among the whole population.

Factors	Events/participants	Prevalence, %	Univariate analysis		Multivariable analysis	
			OR (95% CI)	*P* value	OR (95% CI)	*P* value
Age, yrs						
40–49	17/421	4.04	Ref.	<0.0001	Ref.	0.0442
50–59	9/278	3.24	0.80 (0.35–1.81)		1.40 (0.30–6.51)	
60+	30/193	15.54	4.37 (2.35–8.15)		3.32 (0.82–13.44)	
Gender				0.0023		0.0044
Female	36/394	9.14	Ref.		Ref.	
Male	20/498	4.02	0.42 (0.24–0.73)		0.42 (0.24–0.77)	
Education level				0.0001		0.0180
Low	25/205	12.20	Ref.		Ref.	
Moderate	25/492	5.08	0.39 (0.22–0.69)		0.59 (0.31–1.11)	
High	6/195	3.08	0.23 (0.09–0.57)		0.34 (0.13–0.89)	
Diabetes				0.0002		0.0459
No	27/632	4.27	Ref.		Ref.	
Yes	29/260	11.15	2.81 (1.63–4.85)		1.91 (1.04–3.52)	
Working status				0.0016		0.78
Currently working	18/474	3.80	Ref.		Ref.	
Not working	38/418	9.09	2.53 (1.42–4.51)		0.90 (0.42–1.91)	
Nationality				0.17		0.71
Emiratis	33/446	7.40	Ref.		Ref.	
Non-Emiratis	23/446	5.16	0.68 (0.39–1.18)		1.12 (0.61–2.07)	

OR = odds ratio; CI = confidence interval. Stepwise (forward selection) regression model was used to select covariates. The multivariable model only included the statistically significant risk factors (age, gender, education, and diabetes) in the final multivariable model (*P* < 0.10).

## Data Availability

The datasets used and/or analyzed during the current study are available from the corresponding author on reasonable request.
